# Supplementary material for: Identification of photoperiod-induced specific miRNAs in the adrenal glands of Sunite sheep (Ovis aries)
Source: Front Vet Sci. 2022 Jul 22;9:888207. doi: 10.3389/fvets.2022.888207 (PMC9354845; doi:10.3389/fvets.2022.888207)
Supplement: Supplementary file 1 [file Data_Sheet_1.ZIP › Supplementary file/miRDeep2 file.zip!/miRDeep2 file/Know_Structure/SP42A1/oar-mir-25.pdf]

miRBase precursor : oar-mir-25
Total read count : 15873
oar-miR-25 read count : 15824
remaining reads : 49

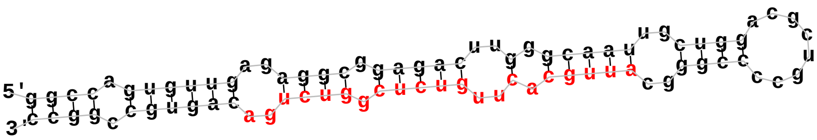

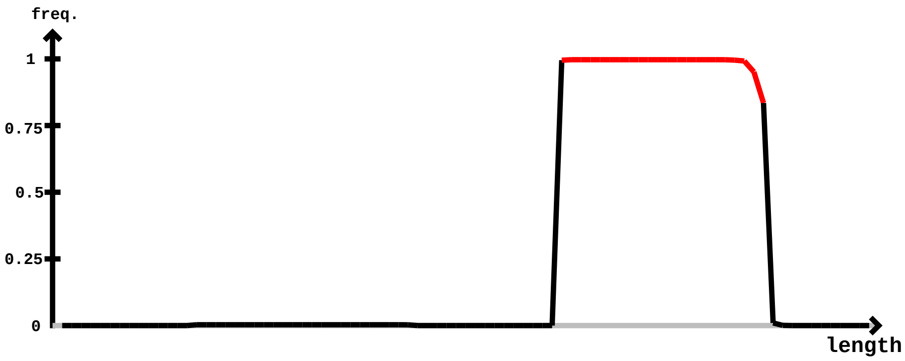

oar-miR-25

```
5'- ggccaguguugagaggcggagacuugggcaauugcuggacgcugccccgggcauugcacuugucucggucugacagugccggcc   -3'    exp
    ((((.((((((..((((.(((((..(.(((((..((((........))))..))))).)..))))).)))).)))))).))))    reads      mm         sample
    .............aggcggagacuugggcaauugc.................................................    5          0          seq
    .............aggcggagacuugggcaauugcu................................................    44         0          seq
    ..................................................gcauugcacuugucucggucug............    2          0          seq
    ...................................................cauugcacuugucucgg................    1          0          seq
    ...................................................cauugcacuugucucggu...............    25         0          seq
    ...................................................cauugcacuugucucgguc..............    52         0          seq
    ...................................................cauugcacuugucucggucu.............    649        0          seq
    ...................................................cauugcacuugucucggucug............    1845       0          seq
    ...................................................cauugcacuugucucggucuga...........    13063      0          seq
    ...................................................cauugcacuugucucggucugac..........    146        0          seq
    ...................................................cauugcacuugucucggucugaca.........    11         0          seq
    ....................................................auugcacuugucucggucug............    4          0          seq
    ....................................................auugcacuugucucggucuga...........    26         0          seq
```
